# Newmark displacement data for low to moderate magnitude events in the Betic Cordillera

**DOI:** 10.1016/j.dib.2020.106097

**Published:** 2020-07-31

**Authors:** José Delgado, Julio Rosa, José A. Peláez, Martín J. Rodríguez-Peces, Jesús Garrido, Meaza Tsigé

**Affiliations:** a Dpto. Ciencias de la Tierra y Medio Ambiente, Universidad de Alicante, Ap. Correos 99, 03080 Alicante, Spain; b Dpto. Física, Ingeniería de Sistemas y Teoría de la Señal, Universidad de Alicante, Ap. Correos 99, 03080 Alicante, Spain; c Dpto. Física, Campus Las Lagunillas, Universidad de Jaén, 23071 Jaén, Spain; d Dpto. Geodinámica, Estratigrafía y Paleontología, Universidad Complutense de Madrid, C/José Antonio Novais 12, 28040 Madrid, Spain; e Dpto. Ingeniería Civil, Campus Fuentenueva, Universidad de Granada, 28071 Granada, Spain

**Keywords:** Earthquake-induced landslides, Hazard maps, Newmark displacement, Low to moderate magnitude, Betic Cordillera

## Abstract

Land-use decisions in relation to seismic-induced landslide hazard are usually made through the preparation of hazard maps. The rigid-block method is probably the most used for this purpose. Under this method, Newmark displacement is computed for each slope unit and this displacement is used as a guide for establishing categories of hazard. At present, most relations used for computing Newmark displacement are established from moderate-to-high magnitude earthquakes (Mw ≥ 6.5). This data article provides Newmark displacements computed from accelerograms recorded in the Betic Cordillera for low-to-moderate magnitude earthquakes (Mw = 3.5–6.3). Records come from the Spanish Strong Ground Motion database (Instituto Geográfico Nacional). Newmark displacements were computed focusing on yield accelerations frequently recorded in such scenarios (0.02, 0.03, 0.04, 0.05, 0.06, 0.08 and 0.10), although higher accelerations were also considered (0.125, 0.15, 0.20, 0.25 and 0.30 g's). These data are useful for the study of the hazard in seismic scenarios of low-to-moderate magnitude, very frequent in practice. These data have been used in the study by Delgado et al. [1].

**Specifications Table**

**Table:** 

**Subject**	Earth and Planetary Sciences
**Specific subject area**	Engineering Geology, Seismically induced landslides
**Type of data**	Table (Excel file)
**How data were acquired**	Accelerograms were recorded and provided by the Spanish Strong Ground Motion Network, operated by the Instituto Geográfico Nacional (IGN).
	Accelerograms were processed using SeismoSignal software [2].
	Newmark displacements were computed in accordance with previously published algorithms [3].
**Data format**	Table of earthquakes (‘Events’).
	Table of records (‘Records’). It includes raw data about event, recording station (name, code, coordinates, epicentral distance and site conditions) and processed data (ground motion severity and Newmark displacements).
	Table of displacements (‘Dn’) includes raw data (magnitude, depth and epicentral distance of station) and processed data (peak ground acceleration, peak ground velocity, Arias intensity, yield or critical acceleration and Newmark displacement).
**Parameters for data collection**	Only accelerograms recorded during earthquakes with magnitude above 3.5 were considered from the whole dataset of accelerograms recorded by the Spanish IGN. Additionally, only records with peak ground acceleration ≥ 0.02 g were processed.
**Description of data collection**	Newmark displacements were computed for processed accelerograms. Processing consisted of baseline correction and bandpass filtering (0.1–25 Hz). For each corrected accelerogram, yield acceleration was set iteratively to 0.02, 0.03, 0.04, 0.05, 0.06, 0.08, 0.10, 0.125, 0.15, 0.20, 0.25 and 0.30 g's, and the corresponding Newmark displacement was computed.
**Data source location**	Strong Ground Motion stations located in the Betic Cordillera (S and SE Spain) [4].
**Data accessibility**	With the article
**Related research article**	Author's name: José Delgado, Julio Rosa, José A. Peláez, Martín J. Rodríguez-Peces, Jesús Garrido, Meaza Tsigé
	Title: On the applicability of available regression models for estimating Newmark displacements for low to moderate magnitude earthquakes. The case of the Betic Cordillera (S Spain)
	Journal: Engineering Geology
	DOI: https://doi.org/10.1016/j.enggeo.2020.105710.

**Value of the Data**

• Available regression models for estimating Newmark displacements were obtained from data of earthquakes of moderate to high magnitude (Mw > 6.0–6.5). Data provided in this paper may complement to existing datasets of Newmark displacements.
• Data are especially valuable to those researchers working in areas of moderate-to-low magnitude seismicity and interested in evaluating hazard related to seismic-induced landslides.
• Data may be merged into existing datasets to build a complete one, or used directly to develop specific empirical models for estimating Newmark displacement relationships.

## 1 Data description

Dataset includes three different tables. In the first table (‘Events’), a list of earthquakes is presented. It contains general information (location, magnitude, intensity, etc.) about the events from which seismic data are used and presented in the other tables. The first column of this table is an index used to link earthquake data in this table with data in the ‘Records’ table.

The second table (‘Records’) lists the accelerograms used to compute Newmark displacements. It repeats part of the information already presented in table ‘Records’ (Date, Time and Name of event) and also contains station name, station code, its location (coordinates) epicentral distance to event, and site conditions description and classification (according to EC8 seismic building code) at the recording site. Additionally, name of the file containing raw acceleration data is given for any interested researchers. For each horizontal component, there is information about severity of ground motion (peak ground acceleration –PGA–, peak ground velocity –PGV–, and Arias Intensity –Ia–) computed after processing raw acceleration data. The first column in this table is an index that refers to the earthquake (Table ‘Events’). In this way, each record may be easily related to the corresponding earthquake. The second column in this table (‘#_Record’) is also an index used to relate the record with displacements presented in the third table of the file (‘Dn’).

Finally, the third table in this file (‘Dn’) contains the Newmark displacements computed from accelerograms presented in table ‘Records’. The first column in this table is the index that points to the record, so as to relate displacements with the corresponding accelerogram. This table contains raw data already presented in table ‘Records’ (magnitude of event, focus depth and epicentral distance) and processed data (PGA, PGV, Ia and Newmark displacement computed for a given yield acceleration, also specified). This table contains data already presented in table ‘Records’ but presented in a column format (instead of row-formatted data), so it is more suitable as input for any software/routine of statistical analysis.

## 2 Experimental design, materials, and methods

To study the applicability of existing empirical regression models used when estimating Newmark displacements for moderate-to-low magnitude earthquake scenarios (Mw < 5.5), the whole dataset of accelerograms of the Spanish Strong Ground Motion database, operated by the Instituto Geográfico Nacional (IGN) [4], has been used. This database contains more than 1900 records. Earthquake magnitudes range from 1.2 (mbLg) to 6.3 (Mw). Raw accelerogram data are available from this organization.

**Fig. 1:**
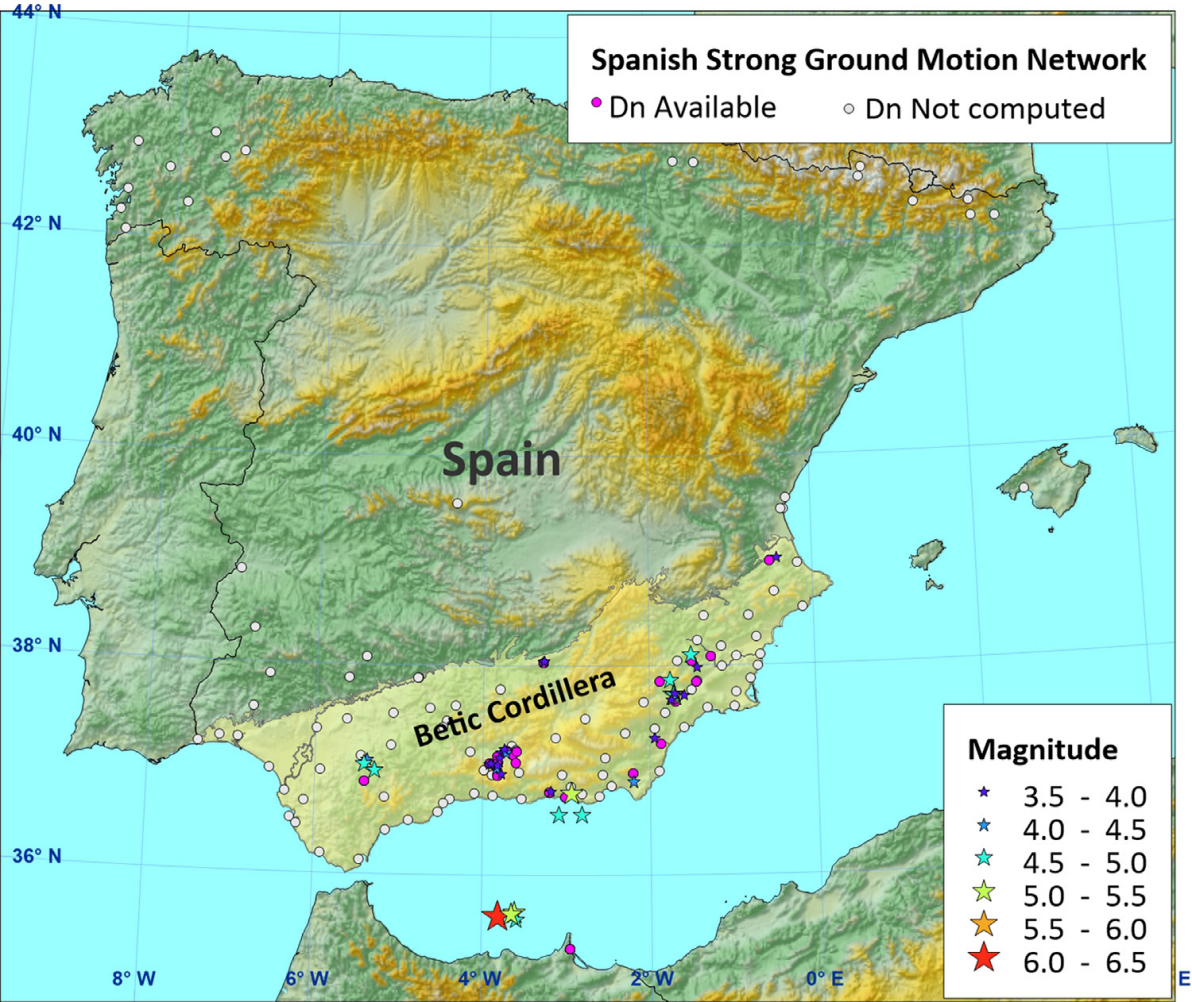
Location map showing the epicentral location of events (Table ‘Events’) and stations (Table ‘records’) used for computing Newmark displacements.

For the dataset presented here, those accelerograms recorded during earthquakes with a minimum magnitude of 3.5, having a minimum PGA of 0.02 g, and recorded at stations located in the Betic Cordillera (S and SE Spain, Fig. 1), have been selected. Lower bound of magnitude is of the order of smaller events known to have triggered small-sized instabilities [5], while the threshold in acceleration is in agreement with the minimum values of PGA required to trigger shallow small-size disrupted landslides in recent earthquakes [6, 7]. With these requirements, a final dataset consisting in 33 earthquakes and 89 records was available for computing displacements.

Once they had been defined, the accelerogram records useful for computing Newmark displacements were processed. First, they were baseline corrected and bandpass filtered (0.1–25 Hz) with a fourth order Butterworth filter. This processing was done through SeismoSignal software [2]. Then, for each record, displacements were computed for both positive and negative polarities considering the largest value for the analysis [8]. Displacements computed from orthogonal components recorded at the same station were considered as separate data [8].

Due to the interest is obtaining Newmark displacements useful for moderate-to-low magnitude scenarios, critical accelerations (ky) used for computing displacements focus on the low range of accelerations (0.02, 0.03, 0.04, 0.05, 0.06, 0.08 and 0.1 g), values commonly found in such seismic scenarios. Nevertheless, higher ky values (0.125, 0.15, 0.2, 0.25 and 0.3 g) were also computed for those records that allowed it.

For the computing of displacements, the algorithm proposed by Jibson [3] was chosen. This algorithm computes Newmark displacements by double integration of those parts of the accelerogram record greater than ky. It takes into account inertia of potential soil mass and, for each pulse of acceleration above ky, computes the velocity (and displacement), and continues integrating accelerations even when they are lower than ky, just until the velocity is zero. A program written in Visual Basic including this algorithm was implemented to automatically compute displacements for all records and ranges of ky.

## Declaration of Competing Interest

The authors declare that they have no known competing financial interests or personal relationships which have, or could be perceived to have, influenced the work reported in this article.

## Appendix Supplementary materials

Image, application 1
